# Professional medical writing support and the reporting quality of randomized controlled trial abstracts among high-impact general medical journals

**DOI:** 10.12688/f1000research.12268.2

**Published:** 2017-09-14

**Authors:** Ira Mills, Catherine Sheard, Meredith Hays, Kevin Douglas, Christopher C. Winchester, William T. Gattrell

**Affiliations:** 1 PAREXEL International, Hackensack, NJ, 07601, USA; 2 Department of Archaeology and Anthropology, University of Bristol, Bristol, BS8 1TH, UK; 3 Department of Medicine, Uniformed Services University, Bethesda, MD, 20814, USA; 4 School of Medicine, Pharmacy and Health, Durham University, Stockton on Tees, TS17 6BH, UK; 5 Research Evaluation Unit, Oxford PharmaGenesis Ltd, Oxford, OX13 5QJ, UK; 6 Oxford Brookes University, Oxford, OX3 0BP, UK; 7 Ipsen Pharma, Abingdon, OX14 4RL, UK

**Keywords:** randomized controlled trials, medical writing, CONSORT guidelines, abstracts, adherence, adverse events, funding source

## Abstract

**Background**: In articles reporting randomized controlled trials, professional medical writing support is associated with increased adherence to Consolidated Standards of Reporting Trials (CONSORT). We set out to determine whether professional medical writing support was also associated with improved adherence to CONSORT for Abstracts.

**Methods**: Using data from a previously published cross-sectional study of 463 articles reporting randomized controlled trials published between 2011 and 2014 in five top medical journals, we determined the association between professional medical writing support and CONSORT for Abstracts items using a Wilcoxon rank-sum test.

**Results**: The mean proportion of adherence to CONSORT for Abstracts items reported was similar with and without professional medical writing support (64.3% vs 66.5%, respectively; p=0.30). Professional medical writing support was associated with lower adherence to reporting study setting (relative risk [RR]; 0.40; 95% confidence interval [CI], 0.23–0.70), and higher adherence to disclosing harms/side effects (RR 2.04; 95% CI, 1.37–3.03) and funding source (RR 1.75; 95% CI, 1.18–2.60).

**Conclusions**: Although professional medical writing support was not associated with increased overall adherence to CONSORT for Abstracts, important aspects were improved with professional medical writing support, including reporting of adverse events and funding source. This study identifies areas to consider for improvement.

## Introduction

Prior studies demonstrate low levels of adherence to Consolidated Standards of Reporting Trials (CONSORT) guidelines ^1, 2^, as well as CONSORT for Abstracts ^3, 4^ in reporting randomized controlled trials (RCTs). Professional medical writing support, correctly acknowledged, is endorsed by Good Publication Practice (GPP3) ^5^, and its prevalence increased between 2001/2002 and 2009/2010, with a reported doubling to nearly 35% of industry-sponsored studies ^6^. Professional medical writing support is associated with increased adherence to CONSORT in articles reporting RCTs; in a sample of open-access journals, the number of articles that completely reported ≥50% of the studied CONSORT items was significantly higher with professional medical writing support (39%) than without professional medical writing support (21%; p<0.05) ^7, 8^.

The purpose of this study was to determine whether professional medical writing support was also associated with improved adherence to CONSORT for Abstracts by analyzing a published dataset from five high-impact general medical journals with overall variable and incomplete adherence ^9^.

## Methods

We examined data from a published cross-sectional study of 463 articles reporting RCTs ^9^. The RCTs were published between 2011 and 2014 in five top medical journals: *The New England Journal of Medicine*, *Annals of Internal Medicine*, *The Lancet*, *The BMJ*, and *JAMA*. We determined the association between professional medical writing support and the reporting of CONSORT for Abstracts items ^10^ ( Table 1) using a Wilcoxon rank-sum test. One author (CS), who was blinded to the CONSORT for Abstracts scores using de-identified original dataset outputs, identified articles as being prepared with professional medical writing support using automated searching of the full text article followed by manual review if they acknowledged the involvement of one of the following: medical writer, medical writing, writing services, writing assistance, editorial assistance, or editorial support. The context of these terms was also examined.

**Table 1.** CONSORT for Abstracts checklist items ^10^, with descriptors by Hays *et al*. ^9^ as used for this post hoc analysis.

CONSORT for Abstracts items		
Abstract evaluation checklist	Yes	No
**Title**		
1. Do the authors state explicitly in the title that the participants were **randomly assigned** to their comparison groups?		
**Trial design**		
2. Is the type of randomized controlled trial described (e.g. parallel group, cluster randomized, crossover, factorial, superiority, noninferiority, or some other combination of these)?		
**Methods**		
**Participants**		
3. Is there a clear description of the eligibility criteria for trial participants (e.g. inclusion and/or exclusion criteria)? Can the reader reasonably describe the study population and assess the generalizability of the trial?		
4. Is there a clear description of the setting of the participants studied (i.e. primary/secondary/tertiary care center, government health clinic, community clinic; level of care provided at study site)? This does not have to mention geographic setting (i.e. country).		
**Intervention**		
5. Are essential features of the experimental and comparison interventions described, including details about the interventions (e.g. dose, route of administration, duration of administration, surgical procedure, or manufacturer of inserted device)? The abstract must give details about the intervention to help the reader quickly assess the validity of the study.		
**Objective**		
6. Is there a clear statement of the specific objective or hypothesis addressed in the trial? Either a clear statement is made or it is not.		
**Outcome**		
7. Do the authors explicitly state the **primary** or **main** outcome for the trial and when it was assessed (e.g. the time frame over which it was measured)?		
**Randomization**		
8. Do the authors clearly describe the method for **random sequence generation** in assigning participants to interventions?		
9. Do the authors clearly describe the method of **allocation concealment**?		
**Blinding**		
10. Is the study “blinded” or “masked” to group assignment?		
11. Does the abstract specify who was “blinded” or “masked” (e.g. participants, caregivers, those assessing outcomes, data analysts)?		
**Results**		
12. Is the number of participants **randomized** to each group stated?		
13. Is the number of participants **analyzed** for each group stated?		
14. Is the primary outcome result for each group stated?		
15. Is there an estimated effect size and precision (i.e. are confidence intervals given)?		
16. Is there an explicit statement of harms or side effects, or an explicit statement of their absence? Harms and side effects do not have to be specifically labeled as such. Abstracts can satisfy this criterion by stating specific outcomes that are not the primary objective of the study and could reasonably be considered a harm or side effect of the intervention or treatment. This is typically stated in the last sentence of the results.		
**Conclusions**		
17. Is the interpretation of the trial clearly stated?		
**Registration**		
18. Is the trial registration number and trial register stated?		
**Funding**		
19. Is the source of funding for the trial stated?		

Mean proportions of CONSORT for Abstract adherence with and without professional medical writing support was compared using a Wilcoxon rank-sum test, and then tested with additional effect of variable journal adherence using an analysis of variance (ANOVA). The relative risk (RR) and 95% confidence interval (CI) for each item’s adherence with and without professional medical writing support were calculated using the command “oddsratio” in the R package *fmsb* 0.5.2 ^11^. All statistical analyses were performed in R 3.2.2 ^12^.

## Results

**Figure 1.**
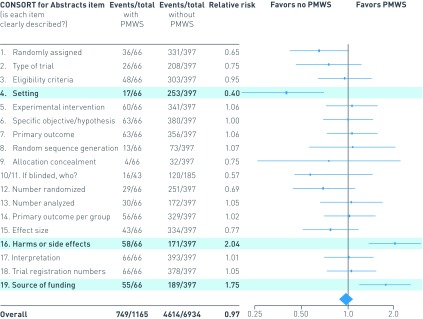
Reporting of CONSORT for Abstracts items in articles with and without acknowledged professional medical writing support. CONSORT, Consolidated Standards of Reporting Trials; PMWS, professional medical writing support. Significant associations are shaded.

From the original published dataset of 463 abstracts from RCTs reported in five journals, acknowledged professional medical writing support was observed in 66 articles (14.3%). Two articles identified in the automated search were excluded on manual review, one of which stated ^13^, “there was no writing assistance from anyone who is not listed as an author,” and the other ^14^, “the Writing committee drafted the report… without editorial assistance.” The mean proportion of CONSORT for Abstracts items reported in articles with (n=66) and without (n=397) professional medical writing support was 64.3% versus 66.5%, respectively; p=0.3044 (Wilcoxon rank-sum test). This difference remained nonsignificant when journal variation in CONSORT for Abstracts adherence was considered (ANOVA, p=0.1347). Overall, reporting of the individual CONSORT for Abstracts items was similar with and without professional medical writing support (RR 0.97; 95% CI, 0.88–1.07) ( Figure 1). However, a lower rate of reporting the study setting (item 4) was observed in articles with professional medical writing support (RR 0.40; 95% CI, 0.23–0.70). Conversely, professional medical writing support was associated with higher adherence to reporting both harms and side effects (item 16) (RR 2.04; 95% CI, 1.37–3.03) and source of funding (item 19) (RR 1.75; 95% CI, 1.18–2.60).

Dataset for reporting of CONSORT for Abstracts items in articles with and without acknowledged professional medical writing support Dataset used per Hays *et al.* ^9^ with the addition of column C for this post hoc analysis by professional medical writing support (yes: 1; no: 0). Click here for additional data file. Copyright: © 2017 Mills I et al. 2017 Data associated with the article are available under the terms of the Creative Commons Zero "No rights reserved" data waiver (CC0 1.0 Public domain dedication).

## Discussion

Although professional medical writing support was not associated with increased overall adherence to CONSORT for Abstracts, important aspects were improved with professional medical writing support, including reporting of adverse events and funding source. These data confirm prior evidence showing that professional medical writing support is associated with improved safety reporting ^15^, and serve to support the important role of professional medical writers in promoting adherence to Medical Publishing Insights & Practices recommendations to improve adverse event reporting in clinical trial publications ^16^. Reporting of funding source was also improved with professional medical writing support, most likely reflecting the emphasis placed on transparency about funding by GPP3 guidelines ^5, 17^. In articles with professional medical writing support, we observed 100% adherence for other important CONSORT for Abstract items, including reporting of the clinical trials registration number (item 18), vital for transparency and study tracking, which has recently been automated using technology such as the TrialsTracker ^18^.

In this post hoc analysis, there was substantial risk of false positives given that separate confidence intervals were computed for 19 different CONSORT items. However, the p value for setting (item 4) was 0.0010, for harms or side effects (item 16) was 0.0004, and for source of funding (item 19) was 0.0055; the first two survive a Bonferroni correction, which would conservatively demand that p<0.0026, and all three pass the less stringent Benjamini-Hochberg procedure. Nevertheless, we were disappointed to see that professional medical writing support was not associated with improvements in reporting of other CONSORT for Abstract items, including specification in the title of the design of the study (item 2) and that it was randomized (item 1), and reporting of the numbers randomized and analyzed (items 12 and 13). Indeed, professional medical writing support was actually associated with worse reporting of one item, study setting (item 4) which may be explained by a lack of prioritization by professional medical writers within the constraints of abstract word count limitations. These areas represent clear areas in which professional medical writers can help further improve the reporting of clinical trials in the abstracts of journal articles. The relatively small number of studies with professional medical writing support is this dataset (n=66) did not allow for further exploration of these data (e.g. type of funding, trial phase, institutions where performed). It would be of interest to determine if similar trends are observed in separate studies of CONSORT for Abstracts adherence.

Although this was a post-hoc analysis, it has the advantage that CONSORT for Abstracts adherence was assessed before our study question was posed. Additionally, the presence of professional medical writing support was assigned by an assessor who was blinded to the CONSORT for Abstracts score. However, in the original study, inter-rater agreement for scoring was 84%, which is suboptimal ^9^. Furthermore, the original dataset was limited to a sample of high-impact journals, and so may not be generalizable to the biomedical literature as a whole. Indeed, in this dataset of high-impact journals, adherence to CONSORT for Abstracts is likely to have been influenced by the journal’s in-house scientific editing; consequently, the impact of professional medical writing support on adherence to CONSORT for Abstracts may be greater in journals without professional in-house editing. These data were potentially confounded by funding source; because professional medical writing support is typically restricted to industry-funded studies, it is possible that the review processes followed by industry ^19^, rather than professional medical writing support *per se*, caused the improvements in reporting that we observed. However, in industry-funded articles, professional medical writing support was associated with a greater than two-fold increase in ≥50% adherence to CONSORT items studied compared with industry-funded articles prepared without this support (38% vs 18%, p<0.05) ^7, 8^. In addition, industry funding alone had no impact on the quality of CONSORT reporting in the absence of professional medical writing support ^7, 8^. Nevertheless, it can be difficult to correctly ascribe the role of the funder from the details provided in manuscripts as, for example, investigator-led studies typically undergo a different review process to those conducted with full industry support ^20^. Finally, although Good Publication Practice guidelines (GPP3) encourage transparency of professional medical writing support ^21^, it remains possible that it was not consistently acknowledged in the studied dataset. In a systematic review of the medical literature, the prevalence of ghostwriting was found to be approximately 5% ^22^. Any unacknowledged professional medical writing support would tend towards the null hypothesis enhancing the confidence in the observed statistical differences.

In summary, although professional medical writing support was not associated with increased overall adherence to CONSORT for Abstracts, important aspects were improved with professional medical writing support, including reporting of adverse events and funding source. Ensuring adherence to reporting guidelines is a complex task, so we believe that there is a role for reporting professionals such as professional medical writers to work with authors and journals, to provide training, writing and reviewing, and thereby improve the quality of reporting of clinical trials.

## Data availability

The data referenced by this article are under copyright with the following copyright statement: Copyright: © 2017 Mills I et al.

Data associated with the article are available under the terms of the Creative Commons Zero "No rights reserved" data waiver (CC0 1.0 Public domain dedication). 

**Dataset 1: Dataset for reporting of CONSORT for Abstracts items in articles with and without acknowledged professional medical writing support.** Dataset used per Hays *et al.* ^9^ with the addition of column C for this post hoc analysis by professional medical writing support (yes: 1; no: 0). doi, 10.5256/f1000research.12268.d172437 ^23^
