# Supplementary material for: Professional medical writing support and the reporting quality of randomized controlled trial abstracts among high-impact general medical journals
Source: F1000Res. 2017 Sep 14;6:1489. Originally published 2017 Aug 16. [Version 2] doi: 10.12688/f1000research.12268.2 (PMC5615774; doi:10.12688/f1000research.12268.2)
Supplement: Peer review history

# Peer review: Referee response for version 2

**DOI:** 10.5256/f1000research.13720.r25988

**Published:** 2017-09-22

**Authors:** Ana Marušić

**Affiliations:** 1 Department of Research in Biomedicine and Health, University of Split School of Medicine, Split, Croatia

The authors have addressed my concerns and comments. Although the information presented is rather obvious and based on a previous study, it is important evidence in the field and is suitable for indexing as a research note.

I have read this submission. I believe that I have an appropriate level of expertise to confirm that it is of an acceptable scientific standard.

# Peer review: Referee response for version 2

**DOI:** 10.5256/f1000research.13720.r25987

**Published:** 2017-09-18

**Authors:** Jackie M. Marchington

**Affiliations:** 1 Caudex, Oxford, UK

I have no further comments. Thank you.

I have read this submission. I believe that I have an appropriate level of expertise to confirm that it is of an acceptable scientific standard.

# Peer review: Referee response for version 1

**DOI:** 10.5256/f1000research.13282.r25067

**Published:** 2017-08-23

**Authors:** Jackie M. Marchington

**Affiliations:** 1 Caudex, Oxford, UK

This is a subanalysis of a previous study detailing the completeness of clinical study reporting in abstracts according to the CONSORT for abstracts checklist. The original publication was restricted to a sample of clinical trial publications in 5 high-ranking general medical journals. This subanalysis investigated differences in adherence to CONSORT for abstracts between papers with or without acknowledged professional medical writing support.

 With respect to the co-authors' previous study, citation of the full paper (ref 8) is sufficient. Citing the poster of the same study is not necessary (ref 7).

 Regarding the methods, it is not clear how automated searching would have identified the presence of professional medical writing support. In this reviewer's experience, that has always required retrieval of the full text article. If the automated search was performed on each full text article, I think it would be clearer to state this.

 In the discussion, the authors point out funding source as a potential confounder of their results. It would perhaps be of interest to examine industry-funded studies written up without acknowledged professional medical writing support as an additional comparator group to determine whether industry funding was driving differences in CONSORT adherence.

 I also have a niggling concern that although GPP3 encourages transparency around the involvement of non-author contributors such as professional medical writers in industry-funded publications, there may be papers in this cohort that used unacknowledged medical writers, papers where a professional writer was included as an author, so not acknowledged as such, or non-industry funded papers where the authors used institutional editorial/writing resource that is not acknowledged. Perhaps the authors could comment on this potential limitation in the discussion.

 Although this is a fairly straightforward subanalysis of a previous study, I think it makes an important contribution to the evidence base about the role of professional medical writers, even though the findings are neutral in terms of adherence to CONSORT for abstracts.

I have read this submission. I believe that I have an appropriate level of expertise to confirm that it is of an acceptable scientific standard.

# Response

**Published:** 2017-09-11

**Authors:** Ira Mills

**Affiliations:** PAREXEL, USA

We thank **Jackie Marchington** for her insight and providing suggestions to improve our paper.

 Although Good Publication Practice guidelines (GPP3) encourage transparency of professional medical writing support, it remains possible that professional medical writing support was not consistently acknowledged in the studied dataset, and cited evidence in the literature of its prevalence. Any unacknowledged professional medical writing support would tend towards the null hypothesis enhancing the confidence in the observed statistical differences.

 As noted by the referee, we have pointed out funding source as a potential confounder of the results and provided discussion as to some of these considerations. The relatively low number of articles with acknowledged professional medical writing support did not allow us to conduct an analysis of professional medical writing support with and without industry support with any statistical validity.

 In our paper to support cited information, we have listed both reference 7 (full publication) and reference 8 (corresponding abstract for a prior congress presentation) as the abstract reported p values not found in the full publication. We have swapped the order of the references to have the full publication cited first.

# Peer review: Referee response for version 1

**DOI:** 10.5256/f1000research.13282.r25066

**Published:** 2017-08-17

**Authors:** Ana Marušić

**Affiliations:** 1 Department of Research in Biomedicine and Health, University of Split School of Medicine, Split, Croatia

This study presents secondary analysis of a study of adherence to CONSORT reporting guidelines for Abstracts. While the original study (Hays et al, 2016) looked at this adherence in a sample of abstracts from several high-profile medical journals, this study performed a subgroup analysis, looking at whether the mention of professional writing assistance was associated with the score for different items on the reporting checklist. The study is thus quite methodologically biased, and it is not clear whether the observed differences are real, or random. For example, the finding that the acknowledgment of professional writing assistance decreases the frequency of adequately reporting the setting of the study is difficult to explain. The potential randomness of the significant findings should be addressed in the Discussion section.

 Making a publication out of the original publication by looking at a single characteristic does not really warrant the publication of the submitted manuscript. It would be preferable if other characteristics were explored in relation to reporting completeness in abstracts, such as the type of funding, phase of the trial, institutions where trials were performed.

 The manuscript is well written and structured, and there are no major comments or suggestions for improvement except the conceptual one described above. In the Methods section, the extraction of data for this study is not fully explained - how many experts extracted the data, how were different terms in the statements on writing assistance interpreted and categorized, etc.

I have read this submission. I believe that I have an appropriate level of expertise to confirm that it is of an acceptable scientific standard, however I have significant reservations, as outlined above.

# Response

**Published:** 2017-09-11

**Authors:** Ira Mills

**Affiliations:** PAREXEL, USA

We thank **Ana Marušić** for her insight and providing suggestions to improve our paper.

 We feel that the data described herein fulfill the criteria for publication as a “Research Note” in F1000 Research, designed to report “single-finding papers that can be reported with one or two illustrations.” Our intention was to conduct a post hoc analysis to specifically examine the role of professional medical writing support on CONSORT adherence as previously described by two of the authors.

 In the discussion, we acknowledge that there was substantial risk of false positives given that separate confidence intervals were computed for 19 different CONSORT items. However, the p value for setting (item 4) was 0.0010, for harms or side effects (item 16) was 0.0004, and for source of funding (item 19) was 0.0055; the first two survive a Bonferroni correction, which would conservatively demand that p<0.0026, and all three pass the less stringent Benjamini-Hochberg procedure.

 We now include a note in the discussion that the limited number of articles with acknowledged professional medical writing support did not allow further exploration of the results (e.g. type of funding, trial phase, institutions where performed) and also mention that it would be of interest to examine whether similar trends are observed in separate studies of CONSORT for Abstracts.

 As described in the original submission, one author performed the data extraction conducting a blinded automated search per the defined terms followed by reexamining for context. We note two articles that were excluded from the analysis by manual review and provide rationale. In one case there was no writing assistance from anyone not listed as an author and in the second case the “Writing committee drafted the report…without editorial assistance.” We have made it clear that this was performed using the full text articles.
